# Oxidative Stress Enhances Rubella Virus Infection in Immortalized Human First-Trimester Trophoblasts

**DOI:** 10.3390/ijms26031041

**Published:** 2025-01-25

**Authors:** Quang Duy Trinh, Kazuhide Takada, Ngan Thi Kim Pham, Chika Takano, Takahiro Namiki, Shun Ito, Yoshinori Takeda, Shoko Okitsu, Hiroshi Ushijima, Satoshi Hayakawa, Shihoko Komine-Aizawa

**Affiliations:** 1 Division of Microbiology, Department of Pathology and Microbiology, Nihon University School of Medicine, Tokyo 173-8610, Japan; takada.kazuhide@nihon-u.ac.jp (K.T.); pham.thikimngan@nihon-u.ac.jp (N.T.K.P.); takano.chika@nihon-u.ac.jp (C.T.); namiki.takahiro75@nihon-u.ac.jp (T.N.); ito.shun@nihon-u.ac.jp (S.I.); takeda.yoshinori@nihon-u.ac.jp (Y.T.); shoko-tky@umin.ac.jp (S.O.); ushijima-hiroshi@jcom.home.ne.jp (H.U.);; 2 Department of Applied Molecular Chemistry, College of Industrial Technology, Nihon University, Chiba 274-0072, Japan

**Keywords:** rubella, pregnancy, CRS, placenta, trophoblast, infection, oxidative stress, ER stress, susceptibility, congenital rubella syndrome, flow cytometry

## Abstract

Rubella infection (RuV) during early pregnancy is a known cause of congenital rubella syndrome (CRS). However, the mechanisms by which the virus crosses the placenta and infects the fetus are not fully understood. It has been known that various kinds of cell stresses can occur during the placenta formation. Previously, we demonstrated that low-glucose-induced endoplasmic reticulum stress could drastically enhance RuV infection in immortalized human first-trimester trophoblast cells. In this study, we investigated the roles of oxidative stress in RuV infection in these cells. Oxidative stress was induced in Swan.71 cells by culturing them in medium containing hydrogen peroxide (H_2_O_2_) in various concentrations and durations (50 µM or 100 µM for 24 h, or 150 µM for 1 h). RuV infection with a clinical strain was performed 24 h post-treatment, and capsid proteins were visualized at 24 and 48 h post-infection (hpi) using flow cytometry (FCM) and fluorescence microscopy (IF), respectively. The findings demonstrated that oxidative stress significantly enhanced RuV infection, as evidenced by FCM analysis, showing a twofold increase in infection rate, and confirmed by IF assay. Additionally, significantly increased intracellular viral replication was observed at 3 dpi. These findings suggest that oxidative stress during early pregnancy may promote the maternal-to-fetal transmission of rubella, contributing to the development of CRS.

## 1. Introduction

Congenital Rubella Syndrome (CRS) is characterized by defects or disorders at birth, such as hearing loss, eye abnormalities, and congenital heart defects. It primarily affects newborns whose mothers were infected with the rubella virus (RuV). RuV belongs to the family *Matonaviridae* and genus *Rubivirus* and is typically associated with a mild, rash-producing, febrile illness in children [1]. It is well established that the majority of CRS cases were born to mothers who were infected with the rubella virus during the first trimester of pregnancy, up to 90% of cases during the first 8 weeks of gestation [2]. However, the mechanisms underlying CRS have not been well understood.

Early pregnancy is characterized by placenta formation. During pregnancy, the placenta plays a critical role in ensuring the fetus’s growth and health. It provides oxygen, nutrients, and necessary elements for fetal development through the maternal blood supply via the spiral artery system. Placenta formation is initiated from trophoblasts, which arise from the trophectoderm of the blastocyte at the end of the first week of conception. In a placenta, many villi are divided into two types, anchoring and floating villi. Each villus contains an outer bilayer of trophoblasts functioning in nutrient exchanges and an inner core containing placental blood vessels that link to fetal circulation. The outer bilayer comprises an outer layer of continuous multinucleated syncytiotrophoblasts (STBs) and an inner layer of mononuclear cytotrophoblasts (CTBs), which can fuse into the outer layer. In anchoring villi, the cytotrophoblasts of the villous tips differentiate into extravillous trophoblasts (EVTs) that migrate out and invade the decidua [3,4,5]. Therefore, CTBs, and especially the EVTs, have a chance to come in contact with the mother’s blood since the first trimester, while the STBs are directly exposed to maternal blood from the second trimester of pregnancy onwards. Consequently, trophoblast cells are considered the first barrier in protecting the fetus from potential infections originating from the mother’s side. Understanding the infection of trophoblasts by RuV is crucial to unraveling the mechanisms underlying transplacental infection of viruses, including CRS.

Trophoblasts are well known for their resistance to harmful pathogens, including viruses. However, while trophoblasts have been shown to resist RuV infection in lab research environments, CRS cases continue to be reported in clinical settings, particularly following rubella epidemics [6,7]. This apparent discrepancy has posed significant challenges in researching the mechanisms underlying CRS.

As mentioned earlier, up to 90% of CRS cases occur in infants born to mothers infected with RuV during the first trimester of pregnancy, a critical period when the placenta is forming. Low concentrations of oxygen, nutrients, and other essential elements may be present during this stage. Trophoblasts are exposed to various cellular stresses, including oxidative stress, during this critical phase of development [8]. To study RuV infection in the first trimester, we utilized the Swan.71 cells, which are representative of CTBs/EVTs during early pregnancy. These cells were derived from the telomerase-mediated transformation of a 7-week cytotrophoblast isolate [9]. They were cultured under an experimental oxidative stress environment to investigate the role of oxidative stress in RuV infection.

Our previous studies showed that first-trimester trophoblasts exhibited low sensitivity to RuV under normal culture conditions [10]. These cells do not express Myelin Oligodendrocyte Glycoprotein (MOG), a cellular receptor for RuV often found in neural cells [11]. Additionally, we demonstrated that endoplasmic reticulum (ER) stress induced by low-glucose conditions drastically enhanced RuV infection in these cells [12]. In line with hypotheses suggesting that cellular stress might influence RuV infection of trophoblasts during early pregnancy, our current study reveals that oxidative stress, experimentally induced by hydrogen peroxide (H_2_O_2_) treatment, also enhances RuV infection in Swan.71 cells, an immortalized human first-trimester trophoblast cell line.

## 2. Results

### 2.1. Expression of Cell Stress Markers by H_2_O_2_

No significant changes in cell viability were observed in cells cultured in a 96-well plate and treated with H_2_O_2_ in various concentrations and conditions: at 50 µM or 100 µM for 24 h, or stimulated at 150 µM for 1 h and then cultured for an additional 23 h. Real-time PCR analysis of RNA extracted from trophoblast cells cultured in a 12-well plate and treated with H_2_O_2_ revealed an elevated expression of NRF2 (nuclear factor erythroid 2-related factor 2) mRNA, indicative of oxidative stress. Additionally, increased mRNA levels of HIF-1α (hypoxia-inducible factor 1α), associated with hypoxia, and CHOP (C/EBP homologous protein), linked to ER stress, were observed alongside the enhanced NRF2 mRNA expression. Nevertheless, no increase in MOG mRNA expression was noted.

### 2.2. Enhancement of RuV Infection Rate in Oxidative-Stressed Trophoblast Cells by FCM Analysis

The Swan.71 cells cultured in a 96-well plate with various H_2_O_2_ concentrations and culture conditions were infected with RuV (clinical isolate) at a multiplicity of infection (MOI) of 5 to 10 to ensure every single cell has a chance to come in contact with at least one viral particle. The flow cytometry (FCM) analysis was then performed at 24 hpi to measure the RuV infection rate in each sample.

**Figure 1:**
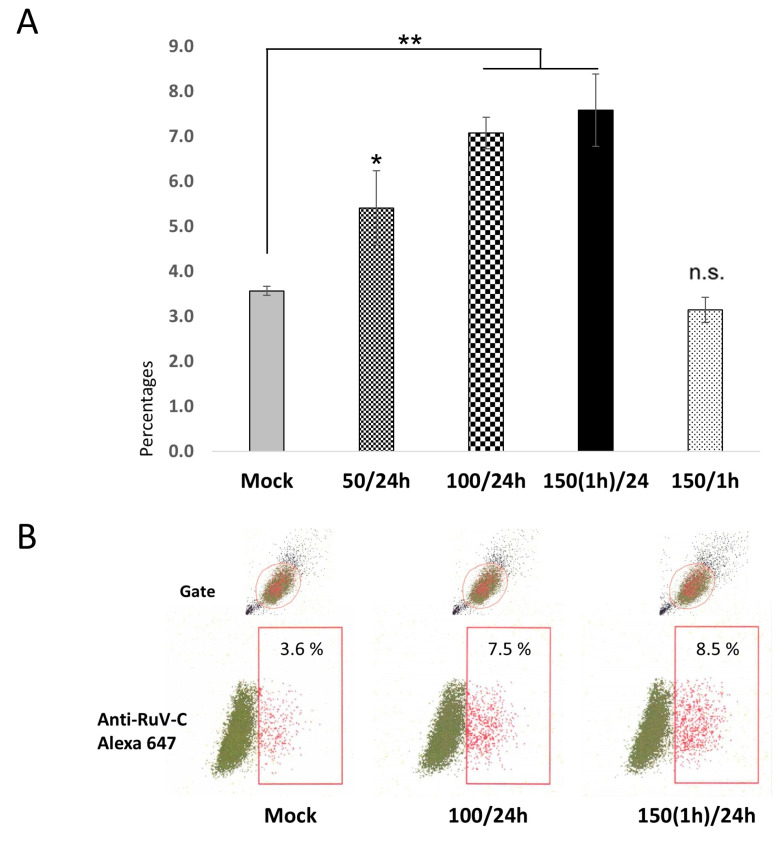
(**A**) **Percentages of the studied trophoblast Swan.71 cells positive for RuV as determined by FCM analysis at 24 hpi.** Trophoblast cells were seeded at a 5 × 10^3^ cells/well density in a 96-well plate. After H_2_O_2_ treatment and RuV infection at an MOI of 5–10, the cells were collected at 24 hpi and labeled with a Live/Dead Fixable Near-IR Dead Cell Stain Kit to exclude dead cells from analysis. Intracellular staining was performed with a mouse monoclonal anti-rubella viral capsid antibody followed by an Alexa-488 conjugated goat anti-mouse IgG (H + L) secondary antibody. Mock-infected cells using a culture medium were used as negative controls. Vero cells infected with RuV performed in parallel were used as positive controls. The results are expressed as the mean of at-least triplicate experiments in each group, and each graph is representative of three independent experiments. Mock: cells cultured in the non-H_2_O_2_ medium; 50/24 h (or 100/24 h): cells were cultured in medium containing 50 μM (or 100 μM) H_2_O_2_ for 24 h before the viral infection; 150(1 h)/24: treated the cells with H_2_O_2_ at 150 μM for 1 h, washed, and cultured for an additional 23 h before the infection; 150/1 h: the infection was performed at 1 h post-treatment with 150 μM H_2_O_2_. * *p <* 0.05, ** *p <* 0.01, n.s: non-significant. (**B**) **Representative images of flow cytometry analysis.** Numbers displayed inside each panel correspond to the percentage of the cells positive for the RuV capsid protein of the parent-gated population.

The results showed that the H_2_O_2_ treatment conditions showed an increased RuV infection in the treated Swan.71 cells, starting with the 50 µM of H_2_O_2_ for 24 h *(p <* 0.05). Compared to the mock-treated cells, the increase in RuV infection turned clearer, with the noted infection rate doubled in trophoblast cells treated with H_2_O_2_ at 100 µM for 24 h, or 150 µM for 1 h and the infection was performed at 24 h post-treatment (*p <* 0.01). However, such an enhancement effect was not observed in the cells treated with 150 µM H_2_O_2_ for 1 h, but the infection was performed right after the treatment (Figure 1).

### 2.3. Enhancement of RuV Infection and Replication in Oxidative-Stressed Trophoblast Cells as Shown by Immunofluorescence Assay and Real-Time PCR

**Figure 2:**
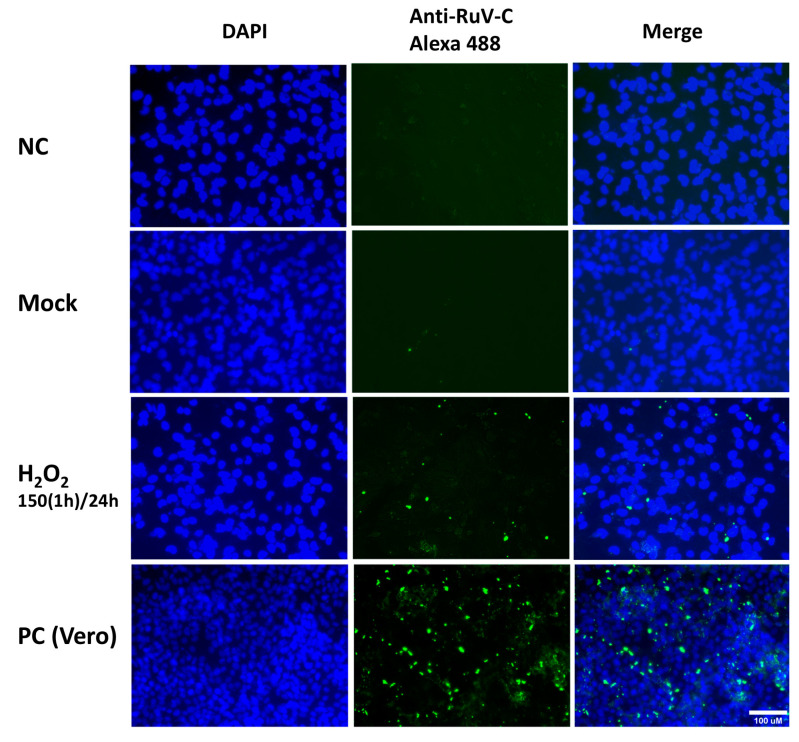
Representative microscopy images of Swan.71 cells infected with RuV under H_2_O_2_-induced oxidative stress condition. The cells were seeded onto 6-well plates containing glass coverslips and cultured for 1 to 2 days before H_2_O_2_ treatments and viral infection. The cells were fixed at 48 hpi and labeled with a mouse monoclonal anti-rubella viral capsid antibody (ab34749, Abcam, Cambridge, UK), followed by an Alexa 488-conjugated goat anti-mouse IgG (H + L) secondary antibody (green). Nuclei were stained with DAPI (blue). RuV-infected Vero cells were used as positive controls. Trophoblast cells mock-infected, incubated with heat-inactivated RuV, or stained with mouse serum were used as negative controls. Images are representative of 3 independent experiments. RuV-C, rubella virus capsid protein. Scale bar: 100 μM. 150(1 h)/24: treated the cells with H_2_O_2_ 150 μM for 1 h, washed, and cultured them for an additional 23 h before viral infection.

At 2 dpi or 3 dpi, an immunofluorescence assay was performed using an antibody to detect the RuV capsid protein, and the cells were visualized under a fluorescent microscope. For the cells treated with H_2_O_2_ using the above-described concentrations and the viral infection step performed at 24 h post-treatment, an increased signal of intracellular localization of the RuV capsid protein was observed, clearly with the H_2_O_2_ treatment at 100 µM for 24 h, or 150 µM for 1 h (Figure 2).

**Figure 3:**
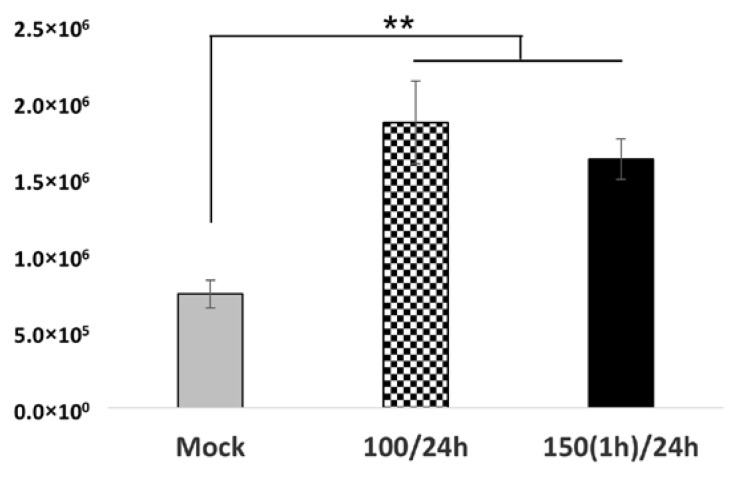
An investigation of viral replication in oxidative-stressed RuV-infected Swan.71 cells. Trophoblast cells were cultured in a 48-well plate, subjected to H_2_O_2_ treatment, and then inoculated with RuV. At 72 hpi, the cells were collected for RNA extraction and real-time PCR. The results are expressed as the mean of triplicate experiments in each group. Mock: cells cultured in the non-H_2_O_2_ medium; 100/24 h: cells were cultured in medium containing 100 μM for 24 h before the viral infection; 150(1 h)/24: treated the cells with H_2_O_2_ at 150 μM for 1 h, washed, and cultured for an additional 23 h before the infection. ** *p <* 0.01.

Additionally, real-time PCR performed on RNA extracted from cells collected at 3 dpi showed significantly increased viral copies in the cells treated with H_2_O_2_ at 100 µM for 24 h or 150 µM for 1 h before the viral infection at 24 h post-treatment, corroborating the experimental findings (Figure 3).

## 3. Discussion

Oxidative stress plays a significant role during pregnancy, particularly in the first trimester, when rapid cellular and tissue remodeling is crucial for placental development. This stress arises from an imbalance between the production of reactive oxygen species and antioxidant defenses, worsened by the high metabolic demands of trophoblast cells and the unique microenvironment of early pregnancy, characterized by low oxygen levels, fluctuating nutrient levels, and changes in the extracellular matrix. While these conditions are vital for placental development, they also contribute to cellular stress, including oxidative stress [8]. Oxidative stress is implicated in pregnancy complications such as preeclampsia, intrauterine growth restriction, and miscarriage, highlighting its critical impact on both maternal and fetal health [13,14,15,16]. Recent studies revealed that there is a mutual role between cell stress and microbial infections, especially in viral infections [17].

It is well known that culturing trophoblasts in artificial conditions does not replicate the uterine environment during early pregnancy. The biological differences between trophoblasts cultured in vitro and in the uterus may explain why trophoblast cells in laboratory settings exhibit resistance or low sensitivity to RuV, while clinical observations showed that up to 90% of CRS cases occur in infants born to mothers who were infected with RuV during the first trimester [2].

This study found that oxidative stress promotes RuV infection in human first-trimester trophoblast Swan.71 cells. In addition, the enhancement effect was not observed in the cells treated with 150 µM H_2_O_2_ for 1 h, and the infection was performed right after the treatment. The findings suggest that downstream factors of the oxidative stress response might induce the above effects rather than the direct effects of oxidative stress.

Regarding viral entry, MOG is recognized as a receptor for RuV in the neural system and is rarely found in other cells or tissues, including trophoblasts [18]. In this study, treatment with H_2_O_2_ did not increase the expression of MOG mRNA, suggesting that other factors arising from oxidative stress, such as downstream proteins, moonlighting proteins, or other related factors, may contribute to or be involved in this phenomenon.

An increase in mRNA expression of HIF-1α, a hypoxia marker, and NRF2 raises a question of whether these two proteins are involved directly in RuV entry, serving as a cellular receptor and replication in the stressed cells. Although such direct effects were not observed in published studies, NRF2 and HIF-1α are involved in the infection process of various viruses, including Zika virus and SARS-CoV-2, offer a reservoir for hepatitis B virus survival in vivo, and are involved in the replication of the human cytomegalovirus and HIV [19,20,21,22,23,24,25,26]. Therefore, the roles of these two proteins in RuV infection should be clarified in future studies.

It has been established that there is an interplay of cell stress response pathways, where a single factor or stimulus can trigger various cellular stress responses [27,28]. Lack of oxygen, for instance, can induce oxidative stress and lead to ER and mitochondrial stress, affecting overall cell metabolic activities. In this study, we also observed increased mRNA expression of CHOP, a marker of ER stress, along with enhanced NRF2 mRNA expression. Therefore, further studies should be carried out to clarify the interplay roles of oxidative stress and other cell stress responses in the enhancement of RuV infection in the H_2_O_2_-induced oxidative stressed Swan.71 cells.

Building upon previous findings that glucose stress enhances RuV binding, infection, and replication in trophoblast cells [12], our findings reinforce the hypothesis that cell stress plays a critical role in RuV infection in these cells. This may help explain the observed resistance or low sensitivity in laboratory settings, as in vitro conditions do not fully replicate the actual environment of trophoblast cells during early pregnancy.

Recently, the use of antioxidants in preeclampsia and other conditions potentially leading to poor pregnant outcomes related to oxidative stress has been introduced and widely studied [29,30,31], raising the question of whether their use in this context could be beneficial in reducing the risk of RuV infection. However, as stated earlier, further studies are needed to clarify underlying mechanisms, including the interplay of cell stress responses and animal models using antioxidants, to provide a clear answer to this issue.

In summary, although the underlying mechanisms are not yet fully understood, together with our previous findings on the enhancement of RuV infection by low-glucose stress, the findings align with clinical observations, such as the high incidence of CRS in the first trimester, and were not observed in every pregnant woman infected with RuV. These results highlight the importance of translating in vitro research to explain clinical phenomena and underscore the need for future studies to adopt uterine-mimic culture methods, explant cultures, or in vivo approaches to better reflect the biological conditions of early pregnancy and advance our understanding of RuV infection.

## 4. Materials and Methods

### 4.1. Cell Culture and Virus

The Swan.71 cells used in this study were kindly provided by Dr. Gil Mor (Wayne State University, Detroit, MI, USA). These cells were derived from the telomerase-mediated transformation of a 7-week cytotrophoblast isolate described by Straszewski-Chavez [9]. The cells were cultured in RPMI 1640 medium (Invitrogen, Waltham, MA, USA) supplemented with 10% fetal bovine serum (FBS), 10 mM HEPES (Invitrogen), 0.1 mM nonessential amino acids (Invitrogen), and 1 mM sodium pyruvate (Invitrogen) 100 units/mL penicillin-streptomycin (complete medium). Vero cells were purchased from the Japanese Collection of Research Bioresources Cell Bank and cultured in Dulbecco’s modified Eagle’s medium (DMEM) (Invitrogen) supplemented with 10% FBS and 100 units/mL penicillin-streptomycin. All cells were cultured in monolayers at 37 °C in a humidified 5% CO_2_ incubator.

The clinical RuV strain (3-B1-RK13) was transferred from Kitasato University School of Medicine (Tokyo, Japan). The viral stock solution was prepared by propagating the virus in Vero cells and concentrating the viral particles via ultracentrifugation at 52,000× *g* for 90 min in a Himac CS100GX micro-ultracentrifuge with an S50A rotor (Hitachi Koki Co., Ltd., Ibaraki, Japan). Viral titers were estimated with the TCID50 method or FCM analysis, as described previously [32,33].

### 4.2. Experimental Hydrogen Peroxide-Induced Oxidative Stress Conditions in Trophoblast Cells

Trophoblast cells were seeded in 12-well plates (5 × 10^4^ cells/well), 24-well plates (2.5 × 10^4^ cells/well), or 96-well plates (5 × 10^3^ cells/well) in complete medium and cultured for 1 to 2 days before experimentation. For the oxidative stress inductions, the cells were cultured in serum-free RPMI containing 0.1% bovine serum albumin (BSA) and hydrogen peroxide (H_2_O_2)_, 50 or 100 µM for 24 h, or 150 µM for 1 h. The cells cultured in serum-free normal RPMI (SF medium) were used as a control for the treatment.

### 4.3. Cell Viability Assay

Trophoblast cells were cultured in a 96-well plate and subjected to H_2_O_2_ treatments, as described above. Cell viability was measured using a Cell Counting Kit-8 (Dojindo Laboratories, Kumamoto, Japan) according to the manufacturer’s instructions. The cell density in each well was measured at 450 nm using a microplate reader (iMark Microplate Absorbance Reader, Bio-Rad, Hercules, CA, USA).

### 4.4. Virus Infection

Cells were cultured in 96-well plates (for FCM analysis) or 6-well plates with glass coverslips for IF assays, washed with SF medium at 24 h post-H_2_O_2_ treatment, and incubated with the virus at multiplicities of infection (MOIs) of 5 to 10 for a total of 3 h in a 37 °C, humidified 5% CO_2_ incubator with gentle shaking every 10~15 min during the first hour. The supernatant was removed, the cells were washed, and the medium was replaced with a fresh medium containing 2% FBS. The percentage of cells infected with the virus was determined 24 h post-infection (hpi) by FCM analysis and IF assays at 48 hpi. Negative control cells (mock-infected, infected with heat-inactivated RuV, or not exposed to the primary antibody during the staining procedures) and positive controls using RuV-infected Vero cells were also performed in parallel for comparison.

### 4.5. Immunofluorescence Assay

Cells cultured on glass coverslips in six-well plates were subjected to H_2_O_2_ treatments and then infected with RuV, as described above. At 48 hpi, the supernatant was removed, and the cells were fixed with cold methanol for 5 min, washed with PBS, and incubated with the mouse monoclonal anti-RuV capsid antibody (ab34749, Abcam) for 1 h at RT. Separate negative controls subjected to mock treatment, heat-inactivated RuV inoculation, and staining with normal mouse serum were established. The cells were washed with PBS and incubated with a goat anti-rabbit IgG H&L (Alexa Fluor 488) secondary antibody solution (ab150081, Abcam) for 30 min at RT. The samples were counterstained with 4′,6-diamidino-2-phenylindole dihydrochloride (DAPI; Lonza, Walkersville, MD, USA) for nuclear staining. After washing, the coverslips were mounted with VECTASHIELD Mounting Medium (Vector Labs, Burlingame, CA, USA), and fluorescence images were acquired using a fluorescence microscope (FLoid Cell Imaging Station; Life Technologies, Carlsbad, CA, USA).

### 4.6. FCM Analysis

The cells were collected via detachment medium [RPMI containing 2.9 mM EDTA, 2% FBS, Live/Dead Staining Solution (Live/Dead Fixable Near-IR Dead Cell Stain Kit, Thermo Fisher Scientific, MA, USA)]. After washing with staining buffer (STB, cold PBS containing 5% goat serum and 2 mM EDTA), the cells were fixed and permeabilized using a BD Cytofix/Cytoperm Fixation/Permeabilization Solution Kit (BD Biosciences, San Diego, CA, USA). Intracellular staining was performed with a mouse monoclonal anti-RuV capsid antibody (ab34749, Abcam) for 30 min at RT. The cells were washed and incubated with a goat anti-mouse IgG H&L (Alexa Fluor^®^ 647) secondary antibody (ab150115, Abcam) solution for 30 min at RT. The cells were subjected to FCM analysis after washing and fixation. For each sample, at least 5000 gated events were collected and analyzed on a BD FACSVerse cytometer using BD FACSuite software (version 1.2; BD Biosciences). A separate negative control group without viral inoculation was also established.

### 4.7. RNA Extraction and RT-PCR

Swan.71 cells were cultured in 24-well or 48-well plates and subjected to H_2_O_2_ culture conditions without or with RuV infection, as described above. Total mRNA was extracted using TRIzol reagent (Life Technologies, Tokyo, Japan), and contaminated genomic DNA was removed by treatment with DNAse I (TaKaRa Bio, Inc., Otsu, Japan). Real-time RT-PCR was performed using a One-Step TB Green Prime-Script PLUS RT-PCR Kit (Perfect Real Time; TaKaRa Bio) in a QuantStudio5 Real-Time PCR System (Applied Biosystems, Waltham, MA, USA). The following primer sequences were used: NRF-2, sense, 5′-ACC CTT GTC ACC ATC TCA GG-3′ and antisense, 5′-AGC GGC TTG AAT GTT TGT CT-3′; RuV, sense, 5′-CCA CTG AGA CCG GCT GCG A-3′; antisense, 5′-GCC TCG GGG AGG AAG ATG AC-3′; and peptidylprolyl isomerase A (PPIA), sense, 5′-ATG CTG GAC CCA ACA CAA AT-3′ and antisense, 5′-TCT TTC ACT TTG CCA AAC ACC-3′. The results were analyzed using the delta–delta Ct method.

### 4.8. Statistical Analysis

Analysis of variance was performed to analyze the results. A *p*-value < 0.05 was considered statistically significant, obtained using the Tukey–Kramer test in Statcel 4 software (OMS Publishing, Inc., Tokorozawa, Saitama, Japan). Data were presented as the mean ± SEM.

## Data Availability

Data related to this study are available upon request to trinh.duyquang@nihon-u.ac.jp.
